# Structural Insights for Anti-Influenza Vaccine Design

**DOI:** 10.1016/j.csbj.2019.03.009

**Published:** 2019-03-23

**Authors:** Lifen Han, Cong Chen, Xianlin Han, Shujin Lin, Xiulan Ao, Xiao Han, Jianmin Wang, Hanhui Ye

**Affiliations:** a The United Innovation of Mengchao Hepatobiliary Technology Key Laboratory of Fujian Province, Mengchao Hepatobiliary Hospital of Fujian Medical University, Fuzhou 350025, China; b College of Biological Science and Engineering, Fuzhou University, Fuzhou 350108, China; c NHC Key Laboratory of Systems Biology of Pathogens, Institute of Pathogen Biology, Chinese Academy of Medical Sciences & Peking Union Medical College, China; d Department of General Surgery, Peking Union Medical College Hospital, Beijing, China

## Abstract

Influenza A virus are a persistent and significant threat to human health, and current vaccines do not provide sufficient protection due to antigenic drift, which allows influenza viruses to easily escape immune surveillance and antiviral drug activity. Influenza hemagglutinin (HA) is a glycoprotein needed for the entry of enveloped influenza viruses into host cells and is a potential target for anti-influenza humoral immune responses. In recent years, a number of broadly neutralizing antibodies (bnAbs) have been isolated, and their relative structural information obtained from the crystallization of influenza antigens in complex with bnAbs has provided some new insights into future influenza vaccine research. Here, we review the current knowledge of the HA-targeted bnAbs and the structure-based mechanisms contributing to neutralization. We also discuss the potential for this structure-based approach to overcome the challenge of obtaining a highly desired “universal” influenza vaccine, especially on small proteins and peptides.

## Graphical abstract

**Figure:**
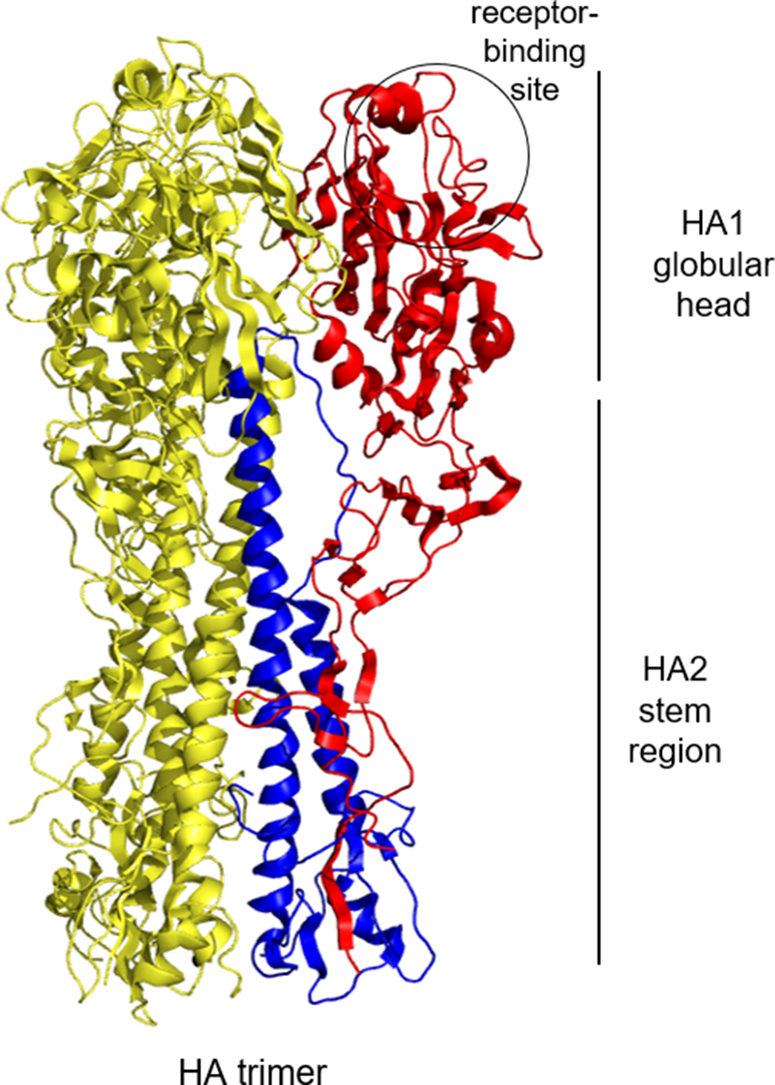

## 1 Introduction

Influenza commonly circulates among humans, causing highly contagious acute respiratory infections in three to five million people worldwide annually [32,45]. In particular, young and elderly individuals are susceptible to severe disease. There were three overwhelming pandemics, namely, the Spanish flu (H1N1) pandemic in 1918 [76,82,98], the Asian flu (H2N2) pandemic in 1957 [1,2], and the Hong Kong flu (H3N2) pandemic in 1968 [3]. Over the past decades, seasonal outbreaks have been caused by influenza A H1N1 and H3N2 subtypes as well as the two lineages of influenza B virus. Influenza virus is a segmented negative-strand enveloped RNA virus that is subject to frequent point mutations within the antigenicity-determining region. These mutations help the virus evade pre-existing immunity, leading to annual epidemics and occasional pandemics. In addition, completely new antigenic strains can emerge from the reassortment of genetic segments and give rise to an unprecedented virus type [71]. For these reasons, influenza viruses pose a constant and significant public health threat.

Influenza virus usually infects and replicates in the epithelial cells lining the surface of the respiratory tract and leads to local inflammation upon human infection. The host immune system targets influenza virus mainly through immune responses that ultimately result in the prevention of virus replication. The main effectors of the humoral immune responses against viral infection are secretory IgA and IgG antibodies. Thus, vaccination provides the most effective strategy to minimize the risk of this virus and represents the only feasible strategy to control a human influenza pandemic at the herd level. In the past, monovalent inactivated vaccines were produced against seasonal influenza strains as quickly as possible upon pandemic emergence. Clinical results showed that more than 90% seroconversion would be acquired in adults immunized with an inactivated vaccine [19,37,42].

**Fig. 1:**
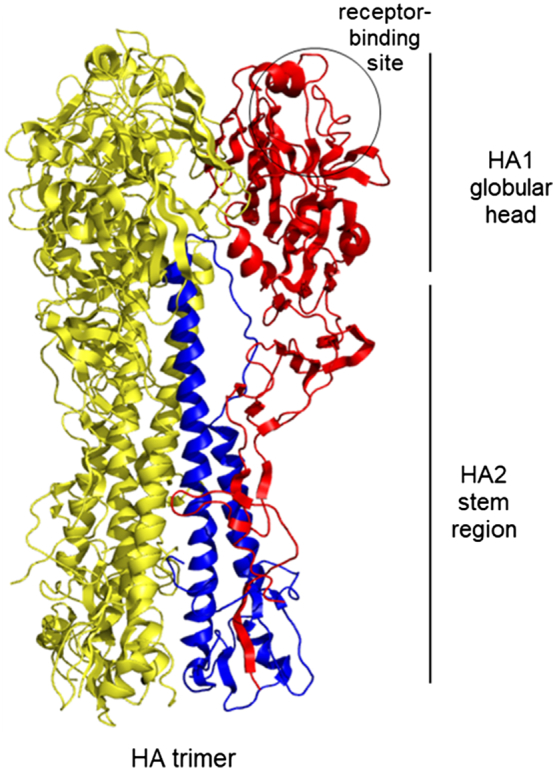
Structure of the influenza HA protein. The representative structure HA (H1 subtype) [Protein Data Bank (PDB) accession number 1RUZ] exists as a trimer on the virion surface and comprised the HA1 globular head (colored red on a single monomer) and the HA2 stem region (blue). The receptor-binding site is circled.

Influenza virus contains eight RNA segments encoding at least 12 proteins (PB2, PB2, PB1-F2, PA, PA-X, HA, NA, NP, M1, M2, NS1, and NS2). The two major surface glycoproteins on the outside of viral particles are hemagglutinin (HA) and neuraminidase (NA), which are essential for viral infection and induce a specific humoral immune response. HA is the most abundant glycoprotein that mediates the virus to attach to the host cell membrane and enter the cell. NA is an enzyme that cleaves the sialoside receptor from the host and enables progeny virus from the infected cells. However, HA greatly outnumbers NA on the virus surface and consequently is the principal target for influenza vaccines. HA presents as a homotrimer, and each of its single-chain monomers is initially synthesized as a precursor polypeptide (HA0) (Fig. 1). Subsequently, the mature HA trimer is cleaved by host cell proteases into two subunits (HA1 and HA2) [41,107], which are linked through a single disulfide bond and numerous hydrogen and hydrophobic bonds. Therefore, HA contains two functional domains: the immunodominant highly variable globular head and a relatively conserved subdominant stem region, which comprise the receptor-binding site (RBS) and the fusion machinery, respectively. A vaccine using the truncated HA region of the influenza A virus has been demonstrated to enhance effectual neutralizing activity and protection against influenza viral challenge [54,59,61,88]. It has been shown that HA proteins play important roles during the immune response to viral infection and are attractive targets for vaccine development. In fact, neutralizing antibodies that target HA either inhibit the binding of influenza virus HA to human cell receptors or prevent low-pH-induced conformational changes to facilitate membrane fusion with a host cell. In general, HA1-targeted antibodies interact with the globular head and show narrow strain specificity, while HA2-targeted antibodies bind to the stem and show broad strain specificity.

Annual vaccination against seasonal influenza plays a valuable role in reducing disease-related mortality and morbidity, but the virus mutating rapidly to evade human immune responses remains problematic. No adequate vaccines so far would likely offer enduring protection against drifting seasonal influenza viruses. More effective vaccines are needed, and the development of broadly cross-neutralizing antibody responses against influenza virus is a critical component of influenza prevention. Due to the constant antigenic drift of the influenza HA, it is crucial to characterize HA at the molecular level and determine how this virus can be interrupted by broadly neutralizing antibodies (bnAbs). In this review, we will discuss current progress on the characterization of influenza virus HA, particularly with the development of more effective influenza vaccines. This review will provide some new insights into future influenza vaccine research and will also stimulate the structure-based design of novel therapeutics.

## 2 Characterization of Influenza Virus HA

**Fig. 2:**
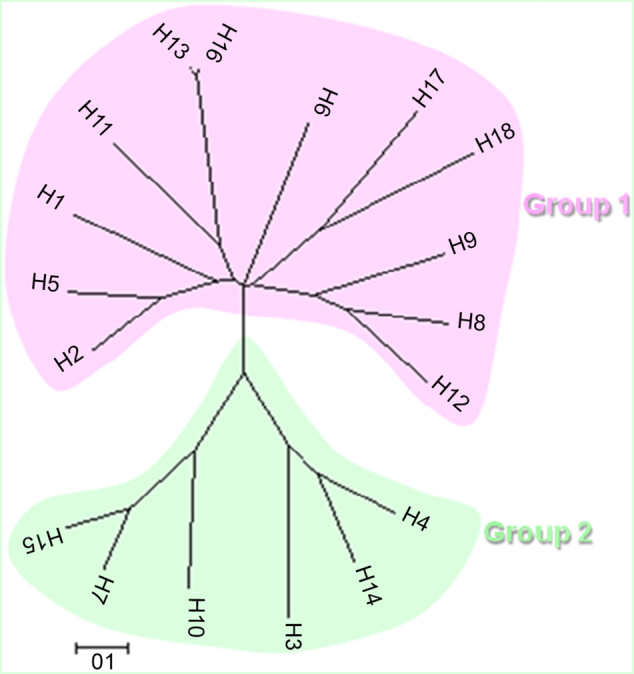
Phylogenetic tree of the influenza A subtypes. The HA are seperated into distinct groups (group 1 in pink and group 2 in green), and each group is further separated to smaller subgroups.

Since the first influenza pandemic occurred in 1918, it took an additional 15 years for an influenza virus to be isolated [93]. Influenza A viruses comprise at least 18 distinct HA subtypes (H1 - H18), whereas influenza B viruses have two separate lineages (the Yamagata and Victoria lineages). The subtypes H1 to H16 are resident in the bird population, whereas two other subtypes (H17 and H18) have been recently discovered in bats [28,103]. Influenza A viruses can also be categorized into two groups based on phylogenetic similarities (Fig. 2): group 1 consists of H1, H2, H5, H6, H8, H9, H11, H12, H13, H16, H17 and H18, while group 2 consists of H3, H4, H7, H10, H14 and H15. HA primarily binds to the cell receptor via 5-*N*-acetylneuraminic acid (sialic acid; SA) to achieve viral attachment and entry [84,109]. Given that antibody blocking prevents virus-to-cell binding, viral HA is the main target for vaccines and protection against influenza virus infection [20,59,95,109].

**Fig. 3:**
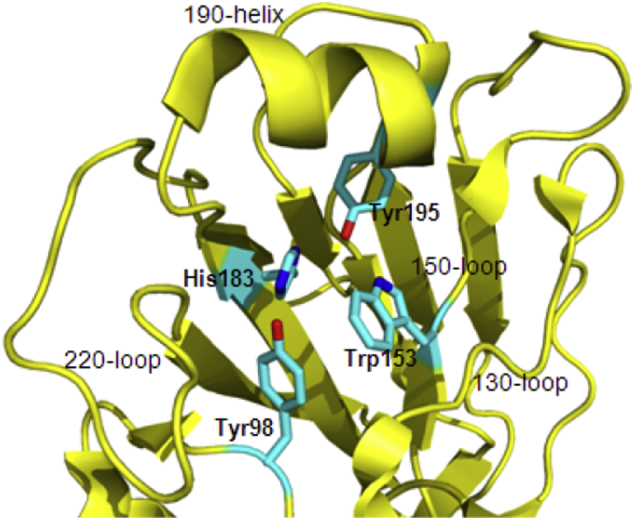
The representative structure HA RBS (H3 subtype) [Protein Data Bank (PDB) accession number 2YP3]. The secondary structure element (the 130-loop, 150-loop, 190-loop and 220-helix) is labeled, and four conserved amino acids are also marked and colored.

The HA of the A/Hong Kong/H3/1968 influenza virus structure was first reported in 1981, and it revealed the first antigen from an enveloped virus [112,113]. Subsequently, the structure of HA-bound receptor complexes with sialic acid analogs was determined in 1988 [109,113]. The structural information for HAs from subtypes of influenza viruses revealed that the HA architecture is highly conserved, but the surface properties and glycosylation patterns extensively differ among influenza subtypes. HA consists of an immunodominant highly variable globular head formed by the HA1 subunit and a relatively conserved subdominant stem region comprising the HA2 subunit and amino acid sequences from the N- and C-termini of the HA1 subunit. The globular domain of HA contains a highly variable amino acid sequence that allows the virus to evade the host immune system. This globular domain consists primarily of the membrane-distal receptor-binding site (RBS), which is responsible for the adsorption of virus to the cell surface, as well as the highly variable immunodominant regions that surround the RBS. The RBS comprises three structural elements (Fig. 3): three loop structures formed by residues 133–138 (the 130-loop), (the 150-loop) and 220–229 (the 220-loop) and a α-helix composed by residues 190–198 (the 190-helix). A number of conserved residues (Tyr98, Trp153, His183 and Tyr195) form the base of the RBS [39,90]. While HA2 constitutes the core of the membrane fusion machinery, and the high diversity of HA is caused by antigenic drift. Antigenic shift can lead to new, re-arranged influenza strains. [85,113].

In fact, several novel influenza vaccine approaches are currently investigated for universal influenza vaccines mainly against both the HA head domain and stem region. Approaches aimed at eliciting broad spectrum immune responses against the HA head domain include computationally optimized broadly reactive antigens (COBRAs). This strategy employs a computational method to produce a consensus sequence of all strains based on the certain HA subtype [34]. It has been encouraging that prototypes COBRA-based vaccines have the ability to elicit protective antibodies against seasonal H1 and H3 isolates and also the pre-pandemic H5 subytpes [12,21,33,34,116]. While, approaches aimed at eliciting universal vaccines against the HA stem region include sequential immunization with heterologous influenza strains and immunization with modified proteins by removing or glycan-masking the globular head. Chimeric HAs can be obtained by shifting the exotic novel HA globular head domains to the HA stalk domains of currently circulating human influenza viruses [40]. Vaccination with headless HAs utilizes constructs that lack the immune-dominant HA head domain [48,106,114]. The current vaccine efficacies do not guarantee complete protection, and thus the need for a broadly protective influenza virus vaccine should be addressed by new approaches.

## 3 BnAbs Specific for the HA Head Domain

Specific bnAbs against influenza virus HA play a critical role in limiting virus replication. Most HA-specific antibody responses upon initial influenza virus infection or vaccination target the globular head domain, which is therefore considered immune-dominant [10,26,67,79,111,123]. To date, the antibody against the HA head domain can be detected in the classical hemagglutination inhibition assay (HI) [43,64]. However, given that the HA head domain is highly variable among different virus strains and undergoes constant changes in antigenic drift, the majority of head specific antibodies are only effective against well-matched circulating virus strains and provide little or no effect against drifted seasonal and possible pandemic viruses [92]. However, a conserved region in the head domain, called the RBS, has been considered to be a fascinating and amenable target of bnAbs. The key amino acids in the HA RBS involved in receptor binding is very highly conserved, but some amino-acid variation can be observed in natural circulating strains in the other regions of the RBS that interact with the other sugar moieties of the receptor. Structure and receptor complexes of the HA has showed how HAs from different subtypes interact with different receptor analogs [31,35,69,86,104]. Antibodies specific for this conserved region exhibit a greater breath than the isolate-specific antibodies normally induced by natural infection and vaccination (Table 1). The monoclonal antibody S139/1 was the first antibody to be described with heterosubtypic reactivity, neutralizing different influenza virus subtypes (H1, H2, H3, H5, H9 and H13 subtypes), which targets the HA head domain and forms a novel conformational epitope adjacent to the RBS [67,119,123]. Crystal structure of S139/1 in complex with Vic75/H3 reveals the structural basis for its neutralization breadth through insertion of its HCDR2 into the RBS [67]. The naturally occurring antibody F045–092 could contact the conserved epitope in the HA head domain and exhibit some activity against H1, H2, H5, and H13 subtypes [65,79]. The F045–092 mainly inserted its 23-residue HCDR3 into the RBS, allowing it to mimic sialic acid to a high degree [65]. CH65 inserted long peptides in the heavy chain directly into the receptor-binding pocket and formed stable complexes, generating strong binding activity in 30 out of 36 H1N1 strains tested [111]. Subsequently, a number of anti-head antibodies have been extensively explored, such as C05 [26,65], CH67 [87], 5 J8 [44], 641 I-9 [86], H2526 [86], 2D1 [58,124], HNIgGA6 [13,14,17], 65C6 [46], H7.5 [105], AVFIulgG03 [97,126], and 100F4 [126] (Table 1). All of these anti-head antibodies contact highly conserved residues of the RBS by inserting its HCDR3, participate in sialic acid receptor binding [87] [13]. Especially, the C05 only utilizes an exceptionally single long HCDR3 and recognizes conserved elements of the RBS by interacting mostly residues on the base of the RBS [26]. Obviously, these anti-head antibodies bind to the conserved RBS pocket by inserting their complementarity-determining loops (CDRs) into the sialic acid receptor pocket, inhibiting the binding of HA to cellular receptors and exhibiting neutralizing breadth. These antibodies mimic the interaction between HA and the influenza virus receptor binding from different directions and have quite different peripheral contacts. Structural analysis of antigen-antibody complexes revealed that the most effective anti-head bnAbs showed remarkably broad neutralizing activity due to their minimal binding footprint on the receptor-binding pocket. The Harrison group reported that antibodies CH65, CH67, 641 I-9, 5 J8 and H2526 only interacted with nine HA residues in the RBS region; however, eight of these residues are either conserved or are human receptor contacts, thus making escape mutations in the HA at these positions unlikely [6,86]. Nonetheless, a small number of antibodies with sialic-acid-like contacts present broad neutralizing breath and inform about possible targets for a universal vaccine for influenza. Besides the RBS, a new conserved site on the HA head of the H1-subtype termed “lateral patch” [83]. This epitope was constant in isolates from 1977 (seasonal) to 2012 (pdm2009). Designated antibody CL6649 could recognize the lateral patch and represents a typical binding mode in most of the circulating H1N1 viruses.

**Table 1:** BnAbs specific for the HA head domain.

BnAbs specific for the HA head	Year	Neutralizing HA subtypes		Reference
		Group 1	Group 2	
FLD21.140	2007	H5	–	Simmons [89]
2D1	2008	H1	–	Yu et al.
S139/1	2009	H1, H2, H5, H9, H13	H3	Yoshida et al.
AVFluIgG01/03	2009	H5	–	Sun et al.
13D4	2009	H5	–	Chen et al. [16]
F045-092	2011	H1, H2, H5	H3	Ohshima et al.
CH65	2011	H1	–	Whittle et al.
5J8	2011	H1	–	Krause et al.
C05	2012	H1, H2, H9	H3	Ekiert et al.
65C6	2012	H5	–	Hu et al.
CH67	2013	H1	–	Schmidt et al.
641 I-9	2014	H1	–	Hong et al.
H2526	2014	H1	–	Hong et al.
HNIgGA6	2015	–	H7	Chen et al.
100F4	2015	H5	–	Zuo et al.
S40	2017	H1	–	Chen et al. [15]
2897	2017	H1	–	Liu et al. [70]
K03.12	2018	H1	H3	McCarthy et al. [74]
CL6649	2018	H1	–	Raymond et al.
H7.5	2019	–	H7	Turner et al.
L4A-14/L3A-44/L4B-18	2019	–	H7	Huang et al. [47]

## 4 BnAbs Specific for the HA2 Stem Region

In contrast, the conservation of the HA stem is restricted to either group 1 or 2 respectively. Due to the increased HA antigenic shift from amino acid alterations, the antibodies specific for the HA stem is more important for the reassortment of different influenza viruses, which might be capable of neutralizing multiple strains of influenza virus. A number of bnAbs specific for the HA stem has been reported, but the eliciting high levels of theses anti-stem antibodies by vaccination remains a challenge due to its poor immunogenicity, mode of immunogenicity, or more restricted access to the HA stem. These antibodies still can be protective and are of interest. The first antibody was isolated in 1983 targeted the HA stem with cross-reactivity in different influenza A virus subtypes but had no detectable neutralizing activity [36]. Ten years later, in 1993, a monoclonal antibody designated C179 was isolated to cross-neutralize multiple subtypes of influenza A viruses H1 and H2 strains [22,80]. The antibody C179 interacted with the HA stem region and inhibited HA fusion activity, resulting in virus neutralization [91]. However, the potential significance of these investigations was not immediately recognized, and the exploration of influenza virus vaccines that target the HA2 stem of circulating influenza virus strains is still in progress.

Two decades later, the first pandemic of the 21st century was caused by a novel influenza strain, H1N1, and a new wave of HA-based influenza virus vaccine approaches started. Within the last decade, a number of human bnAbs specific for the HA stem have been reported (Table 2). A human monoclonal antibody, A06, was derived from a survivor of highly pathogenic H5N1 infection and targeted to the highly conserved stem of HA [24,52,53,96]. This antibody prevented a conformational change of the HA required for viral host cell fusion and thus could neutralize both H1 and H5 subtype influenza viruses. Human monoclonal CR6261 binds to the HA stem with broad heterosubtypic neutralizing activity against diverse influenza A virus subtypes, including H1, H2, H5, H6, H8 and H9 influenza subtypes [24,102]. F10 binds to a highly conserved pocket in the HA stem region and shows remarkable cross-subtype binding and neutralizing potency against influenza virus H1, H2, H5, H6, H8 and H9 subtypes [96]. Recently, several anti-stem antibodies have been reported response to group 1 subtypes, such as Mab3.1, 3E1 and FISW84 [11,108,121]. Interestingly, the monoclonal antibody FISW84 could bind to H1 influenza viruses and its interaction near the junction between the ectodomain and the membrane anchor [11]. It has been shown that these anti-stem antibodies have remarkably broad-range neutralizing properties against most influenza A group 1 viruses but failed to neutralize group 2 subtype viruses. Because of the different site-specific glycosylation site in the HA stem region between group 1 and group 2 influenza subtypes [25,102]. Subsequently, the human monoclonal antibodies CR8020 and CR8043 were isolated with broad neutralizing activity against most group 2 viruses, including H3N2 and H7N7 [25,30]. The antibody SD36 possesses neutralizing activity against influenza A group 2 (H3, H4, H7 and H10) but not group 1 (H1, H2 and H5) [62], which recognized conserved HA stem epitopes with partially overlapped epitopes of bnAbs CR6261 and a lesser extent epitopes of bnAbs CR8020 and CR8043 [24,25,62]. H3v-47 was obtained to neutralize both human and swine H3N2 viruses [8]. Those bnAbs obviously bind to a highly conserved epitope in the HA stem. More recently, some newly identified bnAbs presented surprising properties in terms of their neutralizing potency against both group 1 and group 2 viruses. The mAbs CR9114, F16v3, 39.29, CT149, MEDI8852, 27F3, SD38, 70-1F02 and MD3606 recognized the HA stem of almost all subtypes and exhibited broad heterosubtypic neutralizing activity encompassing both group 1 and group 2 influenza A subtypes [20,23,51,60,62,77,78,120]. In addition, Joyce group designed a number of antibodies (16.a.26, 16.g.07, 31.a.83, 31.b.09 and 56.a.09) from the VRC 310 H5N1 vaccine trial, and those antibodies neutralized viruses from group 1 and 2 subtypes, including H1, H3, H5 and H7, with select antibodies also exhibiting neutralization for H2 and H9 [49]. While, Andrews group designed several antibodies (VRC31504-1D02, VRC 315 02-1F07, VRC 315 13-1B02, VRC 315 27-1C08 and VRC 315 53-1A09) from the VRC 315 H7N9 vaccine trial, which presented a broadly neutralization breath [5]. All of these bnAbs inhibit the membrane fusion activity of HA by preventing the pH-induced conformational rearrangements associated with membrane fusion or limiting viral spread through antibody-dependent cellular cytotoxicity (ADCC), thereby interfering with virus receptor interaction [20,80,96]. It is worth mentioning that several anti-stem antibodies have been evaluated in clinical trials [57,125]. Three monoclonal antibodies CR6261 (ClinicalTrial.gov identifier NCT01992276), CR8020 (NCT01938352) and MEDI8852 (NCT02603952) have been evaluated in hospitalised patients with influenza, demonstrating a well tolerated in phase I clinical trials, and patients are currently recruited for a phase IIa stage [29,72,99]. MHAA4549A (NCT01877785 and NCT02284607) and VIS410 (NCT02045472) have been verified in a phase I and phase IIa study [9,38,68,73,75,100,115]. To date, some trials have been initiated to develop a new generation of vaccines for future pandemic influenza virus therapeutics based on anti-stem bnAbs. However, the main challenge with influenza virus vaccines is the development of vaccines that elicit novel bnAbs not only against currently circulating viruses but also against future antigenically drifted virus strains.

**Table 2:** BnAbs specific for the HA stem region.

BnAbs specific for the HA stem	Year	Neutralizing HA subtypes		VH gene of origin	Reference
		Group 1	Group 2		
C179	1993	H1, H2, H5, H6, H9	H3	VH1–69	Okuno et al.
A06	2008	H1, H5	–	VH1–69	Kashyap et al.
CR6261	2008	H1, H2, H5, H6, H8, H9, H11, H12, H13, H16	–	VH1–69	Throsby et al.
FI6	2009	H1, H2, H5, H6, H8, H9	H3, H4, H7, H10	VH3–30	Wrammert et al.
F10	2009	H1, H2, H5, H6, H8, H9, H11, H12, H13, H16	–	VH1–69	Sui et al.
CR8020	2011	–	H3, H4, H7, H10, H14, H15	VH1–18	Ekiert et al.
FI6v3	2011	H1, H2, H5, H6, H8, H9, H11, H12, H13, H16	H3, H4, H7, H10, H14, H15	VH3–30	Corti et al.
CR9114	2012	H1, H2, H5, H6, H8, H9, H11, H12, H13, H16	H3, H4, H7, H10, H14, H15	VH1–69	Dreyfus et al.
39.29	2013	H1, H2	H3	VH3–30	Nakamura et al.
CR8043	2014	–	H3, H7, H10	VH1–3	Friesen et al.
Mab 3.1	2014	H1, H2, H5, H6	–	VH3–30	Wyrzucki et al.
CT149	2015	H1, H2, H5, H9	H3, H7	VH1–18	Wu et al.
VIS410	2015	H1, H5	H3, H7		Tharakaraman et al.
MEDI8852	2016	H1, H2, H5, H6, H9	H3, H7	VH6–1	Kallewaard et al.
3E1	2016	H1, H5	–	VH4–4	Wang et al.
16.a.26/16.g.07	2016	H1, H5, H9	H3, H7	VH1–18	Joyce et al.
31.a.83		H1, H2, H5, H9		VH3–23	
31.b.09		H1, H5		VH1–18	
56.a.09		H1, H5		VH6–1	
MHAA4549A	2016	H1	H3	–	Gupta et al.
27F3	2017	H1, H5, H6, H9, H11, H12, H13, H16	H3, H7, H10	VH1–69	Lang et al.
VRC 315 53-1A09	2017	H1,H2, H5, H9	H3, H7	VH3–11	Andrews et al.
VRC 315 13-1B02		H1, H5, H9		VH3–48	
VRC 315 27-1C08		H1, H9		VH1–2	
VRC 315 02-1F07				VH3–53	
VRC 315 04-1D02				VH3–53	
FISW84	2018	H1	–	–	Benton et al.
SD36	2018	–	H3, H4, H7, H10	–	Laursen et al.
SD38		H1, H2, H5	H3, H7, H10	–	
MD3606		H1, H9, H12, H13, H14, H15, H16, H17, H18	H3, H7	–	
H3v-47	2018	–	H3	VH1–69	Bangaru et al.
70-1F02	2018	H1, H2, H5, H6, H8, H9, H11, H12, H13, H16	–	VH1–69	Nachbagauer et al.

## 5 Structure-Based Influenza Vaccine Against the HA Head

Conserved viral RBS, at the center of the HA head, is a known target of broadly neutralizing antibodies. The bnAbs against the receptor-binding pocket usually block cell attachment and inhibit viral entry. One of the most striking findings of recent studies is that many of the most potent neutralizing antibodies recognize complex quaternary structures on the surface of viruses. The relatively recent structural information from influenza antigens in complex with bnAbs has provided a framework for interactions at the antigen-antibody interface, accounting for the observed breadth. The structural property of RBS-targeted bnAbs provides a critical understanding of its features based on multiple antibodies-HA complexes and could elicit help inform new broad vaccine immunogens. The anti-RBS bnAbs often use two related strategies to bind to the RBS region, which presents neutralizing activity either because their footprint overlaps with the sialic acid site or because these antibodies exert steric interference [55]. Most bnAbs utilize single-residue insertion near the rim of the sialic-acid pocket to occupy these sites and mimic the activity of the receptor. The dominant mimicry of bnAbs is to precisely interpose a hydrophobic amino acid into the hydrophobic pocket and interact with the acetamide group of the host receptor, sialic acid [13,63,66,86,118]. In addition, some bnAbs mimic this interaction by directly inserting an aspartic acid into this binding pocket [4,44,87,111]. For example, S139/1 inserts its complementarity-determining loop (CDR) H2 into the receptor-binding pocket, which is the endogenous sialic acid binding site [67]. CH65 inserts its CDR H3 domain into the receptor-binding pocket, mimicking the interaction of the physiological receptor [86]. The aspartic acid side chain approaches the location of the sialic acid carboxylate, and the surrounding variable residues of CDR H3 bind to those of the sialic-acid acetamido group. F045–92 and C05 only insert their CDR H3 domains into the receptor-binding site and occupy a very minimal epitope on the HA head, generating strong binding activity [26,65]. One of the challenges of neutralizing influenza at the RBS is the high level of variability of the amino acid sequence in the surrounding receptor-binding pocket. If a bnAb interacts with highly conserved amino acids of the HA RBS, then this antibody may lower the frequency of escape from neutralization and exhibits as an attractive neutralization breadth against most influenza viruses. These observations suggest that the proportion of the buried surface area on the RBS is smaller than the footprint of a typical antibody and hence allows escape through the mutation of nonconserved, peripheral residues.

However, insertions and deletions near the RBS would also restrict the neutralizing potency of the anti-RBS bnAbs. The 133a insertion (between residues 133 and 134) produces a bulge in the 130-loop and mainly exists in the H1 and H5 strains. For example, crystal structure of the S139/1-Vic75/H3 complex reveals that residue 133a produces a localized change in the 130-loop conformation and causes it to bulge and clash into S139/1 [67]. In addition, the side chain of residue 133a interposes into the antibody binding space, which would disrupt this binding. Thus, the 133a insertion appears to negatively influence the binding of S139/1. Another one is the single 158a or double (156a/158b) amino-acid insertions, which are located in the 150-loop and exist in the H4, H6, H7, H10, H14 and H15 strains. The H7.167/H7 HA complex also shows that the insertion of 158a and 158b imparts a unique conformation to the 150-loop in H7 HA, thereby negatively impact the binding of the antibody [101]. Obviously, both insertions abolished the interaction between bnAbs and the receptor-binding pocket [26,67,111]. In addition to these insertions, deletions may also decrease the contact surface and hence the affinity of bnAbs. For example, H7 strains contain an 8-residue deletion in the 220-loop [122].

The binding mode of anti-RBS bnAbs has provided a preferred framework that mimics the key interactions of antibodies with the receptor-binding pocket. The peptide-binding loop, such as HCDR3 from anti-head antibodies, was the basis for the design. For example, small proteins may be rationally designed. The designed high-avidity trimeric protein HSB.2A with a well-characterized small binding domain mainly targets the conserved receptor-binding site and mimics the bnAb C05 binding mode. This molecule exhibits a breadth and potency similar to C05 and protects mice against influenza viruses [94]. Therefore, it is important to design immunogens as small molecules that occupy the space in the RBS, mimic the structure of the epitope that is relevant for antibody neutralization and inhibit influenza virus. In addition, the invariant “lateral patch” also provides a chance for eliciting vaccines recongnizing this conserved epitope.

## 6 Structure-Based Influenza Vaccine Against the HA Stem

Compared to the RBS, the HA stem is highly conserved across all the influenza strains and subtypes and is considered a desirable target for vaccine design. Thus, the anti-stem bnAbs would exhibit a much broader neutralizing breadth against group 1 or group 2 or both group 1 and 2 influenza viruses. BnAbs CR6261, F10 and A06 are specific against influenza A group 1 viruses [53,96,102], whereas FI6v3, 39.29, and CR9114 exhibit neutralizing potency against influenza A groups 1 and 2, and CR9114 also shows reactivity against influenza B viruses [20,23,78]. All of these anti-stem bnAbs bind to a highly conserved hydrophobic pocket in the HA stem. Based on structural information, a number of stem-binding bnAbs have canonical binding modes in which only the heavy chain is involved in the interaction. Among the anti-stem HA antibodies considered to date, the VH1–69 class of antibodies, encoded by a single heavy-chain variable region, is the most well-characterized dominant group, with most donors identified [7,81,117]. The key binding feature of VH1–69 antibodies is a signature motif that encodes hydrophobic residues at Ile53 and Phe54 in CDR H2 and an aromatic Tyr98 in CDR H3 [23,24,52]. These three residues directly interpose into the hydrophobic pocket in the stem region, which provides the ability for high affinity binding and stem recognition. However, the VH1–69-encoded stem-binding bnAbs are more effective against influenza subtype group 1 than group 2. The obstacle that restricts neutralization in group 2 influenza subtypes is due to an N-glycosylation site at HA1 Asn38, which is conserved and only present in group 2 [24,96]. These VH1–69-encoded anti-stem antibodies can be sterically hindered by the oligosaccharide at Asn38. With exception, an antibody CR9114 is able to neutralize both group 1 and group 2 influenza subtypes. It interacts with a similar epitope as group 1 anti-stem bnAbs. Especially, the reorientation of the group-2 specific glycan at HA1 Asn38 adopts an alternative conformation and thus appears essential for CR9114 binding to group 2 influenza subtypes [23]. Recently, three additional multidonor classes of anti-stem bnAbs have been identified that can recognize viruses of different major phylogenetic groups. These antibodies are encoded by VH1–18, VH1–3, and VH6–1 germlines, respectively. In contrast to VH1–69 anti-stem antibodies, these antibodies utilize not only both heavy and light chains for antigen binding but also a junction-encoded residue in CDR H3 to contact the HA stem. Unlike other anti-stem bnAbs, the human monoclonal antibody CR8020 utilizes the heavy chain VH1–18 germline gene and shows broad neutralizing activity against most group 2 viruses [25]. Antibody MEDI8852, whose heavy chain is encoded by the VH1–18 germline gene, has a higher neutralizing potency and breadth [51].

**Fig. 4:**
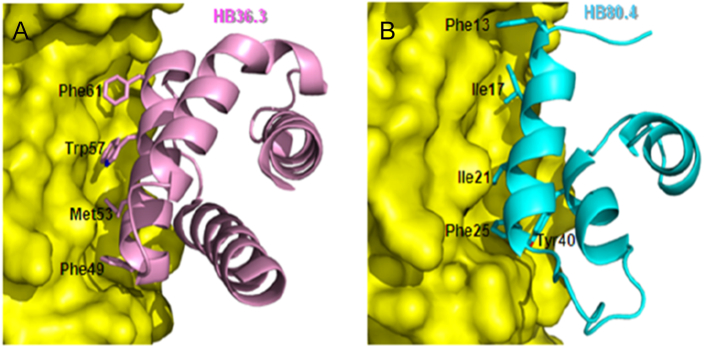
HB 36.3 and HB 80.4 target the HA stem region. (A) and (B) Close-up view of the designed interaction between HB36.3 and HB80.4 and the target site on HA. Contact residues of HB36.3 and HB80.4 are colored and depicted as sticks.

Over the past few years, several antibodies against the stalk domain have been isolated from humans and mice [20,23,24,96]. An improved design of anti-stem bnAbs would consistently elicit heterosubtypic antibody responses and provide a better understanding of human immunity against prepandemic or pandemic influenza. To date, the existence of anti-stem bnAb, which provides a framework for progress, has fueled research in the design of well-defined antigenic constructs leading to a possible universal influenza vaccine. The isolation and characterization of anti-stem bnAbs have raised hopes for a more universal vaccine, as well as the possibility of designing therapeutics. Even more interesting, several small proteins were designed and characterized against the HA stem. The idea was to select small molecules to occupy the space in the HA stem and then exercise an appropriate conformation and configuration to optimize binding. For example, HB80.4 and HB36.3 were engineered de novo on the basis of the paratope of bnAb CR6261 and interact with the HA stem [27,110]. These two proteins use particular amino acid side chains to fill the conserved hydrophobic groove in the HA stem (Phe13, Ile17, Ile21, Phe25, and Tyr40 for HB80.4 and Phe49, Met53, Trp57 and Phe61 for HB36.3) and exhibit a binding mode and neutralization breadth similar to CF6261 (Fig. 4) [27,110]. HB36.3, a variant of HB36 with nine substitutions, affords protection in mice lethally challenged from the 2009 H1N1 pandemic virus [56]. HB80.4, a 51-residue protein, is broadly cross-reactive against all influenza A group 1 HAs and neutralizes H1N1 viruses with a potency akin to those of the best bnAbs [110]. HB1.6928.2.3 represents an excellent alternative to bnAbs for prophylaxis and therapy [18].

Subsequently, several cyclic peptides were designed based on the highly conserved stem region of bnAbs, which could occupy the same hydrophobic grooves in the HA stem region as anti-stem bnAbs. Based on the CDR-interacting loops and framework region 3 of bnAbs CR9114 and FI6v3, a series of linear peptides (P4, P5, P6 and P7) were synthesized and characterized [50]. These peptides target the conserved stem epitope and inhibit low pH-mediated viral membrane fusion. Indeed, these peptides could mimic the binding modes of bnAbs and neutralize influenza virus. The advantageous biological properties of peptides allowed for the rapid development of new small molecular- and peptide-based therapeutics against influenza virus.

## 7 Conclusion Remarks

In recent years, circulating influenza viruses have undergone constant antigenic drift, which acquired immunity against previous infective seasonal strains. It is therefore a strong desire in the field of immunotherapeutics to identify promising vaccine immunogens that can inhibit most influenza viruses. The structural information from influenza antigens in complex with bnAbs has provided a better understanding of how this interaction at the antigen-antibody interface is recognized and how we might induce heterosubtypic immunity to influenza infection. The human bnAbs to influenza virus serves as a template for designing candidate immunogens of a more universal vaccine against influenza viruses against the RBS and HA stem. These bnAbs has provided blueprints for candidate immunogens as therapeutics against influenza virus, and they inspired design of small molecules and peptides to specifically target the RBS on the HA head domain or a region near the fusion peptide on the HA stem region, emulating the neutralization capabilities and mechanisms of the bnAbs. The designed small molecules or peptides should have the capability to interact the conserved RBS or conserved hydrophobic pocket in the HA stem.Although it is clear that many challenges remain, small molecules designed based on the structural characteristics of conserved neutralization epitopes and heterosubtypic antibodies provide another opportunity for therapeutics against influenza viruses and further expand the anti-influenza arsenal. We are hopefully moving closer towards better control of influenza with a universal vaccine that confers long-term immunity.

## Competing Interests

The authors declare no competing financial interests.

## Conflict of Interest

No potential conflict of interest to declare.
